# Distinct molecular phenotype of inflammatory breast cancer compared to non-inflammatory breast cancer using Affymetrix-based genome-wide gene-expression analysis

**DOI:** 10.1038/sj.bjc.6603967

**Published:** 2007-09-11

**Authors:** S Van Laere, I Van der Auwera, G Van den Eynden, P Van Hummelen, P van Dam, E Van Marck, P B Vermeulen, L Dirix

**Affiliations:** 1 Translational Cancer Research Group (Lab Pathology), University of Antwerp, Universiteitsplein 1, Wilrijk B2610, Belgium; 2 Oncology Center, General Hospital Sint-Augustinus, Oosterveldlaan 24, Wilrijk B2610, Belgium; 3 VIB Microarray Facility, UZ-Gasthuisberg, O&N, Leuven 3000, Belgium

**Keywords:** Affymetrix, cell-of-origin subtypes, IBC signature, NF-*κ*B

## Abstract

The present study aims at a platform-independent confirmation of previously obtained cDNA microarray results on inflammatory breast cancer (IBC) using Affymetrix chips. Gene-expression data of 19 IBC and 40 non-IBC specimens were subjected to clustering and principal component analysis. The performance of a previously identified IBC signature was tested using clustering and gene set enrichment analysis. The presence of different cell-of-origin subtypes in IBC was investigated and confirmed using immunohistochemistry on a TMA. Differential gene expression was analysed using SAM and topGO was used to identify the fingerprints of a pro-metastatic-signalling pathway. IBC and non-IBC have distinct gene-expression profiles. The differences in gene expression between IBC and non-IBC are captured within an IBC signature, identified in a platform-independent manner. Part of the gene-expression differences between IBC and non-IBC are attributable to the differential presence of the cell-of-origin subtypes, since IBC primarily segregated into the basal-like or ErbB2-overexpressing group. Strikingly, IBC tumour samples more closely resemble the gene-expression profile of T1/T2 tumours than the gene-expression profile or T3/T4 tumours. We identified the insulin-like growth factor-signalling pathway, potentially contributing to the biology of IBC. Our previous results have been validated in a platform-independent manner. The distinct biological behaviour of IBC is reflected in a distinct gene-expression profile. The fact that IBC tumours are quickly arising tumours might explain the close resemblance of the IBC gene-expression profile to the expression profile of T1/T2 tumours.

Inflammatory breast cancer (IBC) is a clinical diagnosis designated as the T4d category in the TNM classification of the American Joint Committee on Cancer (Singletary *et al*, 2002). It is a distinct clinical subtype of locally advanced breast cancer (LABC), with a particularly aggressive behaviour and poor prognosis (median 3-year survival of about 40%). Clinically, IBC typically presents with rapidly progressive breast erythema, warmth, oedema and induration, often without palpable underlying tumour mass (Kleer *et al*, 2000; Lerebours *et al*, 2005; Dirix *et al*, 2006). Tumour emboli in dermal lymphatics may be apparent on skin biopsy, but in the absence of clinical findings do not indicate IBC (Kleer *et al*, 2000; Low *et al*, 2004; Lerebours *et al*, 2005; Dirix *et al*, 2006). Whether the particular clinical presentation of IBC is a reflection of a specific biologic subtype in human breast cancer remains unclear.

Several studies have been undertaken to gain deeper insights towards understanding the biological characteristics of IBC. At molecular level, overexpression of RhoC GTPase and loss of WISP3 is linked to breast tumours from patients with IBC (Van Golen *et al*, 1999; Kleer *et al* 2002). At biological level, increased angiogenesis in breast tumours from patients with IBC was demonstrated in several independent studies (Colpaert *et al*, 2003; Van der Auwera *et al*, 2004). Using cDNA microarrays, Bertucci *et al* (2004) and Van Laere *et al* (2005) identified gene-expression signatures associated with the IBC phenotype. Using the same expression data sets, both studies demonstrated the presence of the same cell-of-origin breast cancer subtypes in IBC (Bertucci *et al*, 2005; Van Laere *et al*, 2006a), as was originally described for non-IBC (Perou *et al*, 2000). In depth analysis of genes differentially expressed between breast tumour samples from patients with IBC and other breast tumour samples revealed a potential hyperactivation of NF-*κ*B in IBC (Van Laere *et al*, 2005), which was validated and confirmed in an independent study (Van Laere *et al*, 2006b).

The current study presents a follow-up study of our previous cDNA microarray experiments comparing non-inflammatory and inflammatory breast carcinoma using a platform-independent approach. The main focus of this study is to validate our observation that breast tumours from patients with IBC and breast tumours from patients without IBC have a different gene-expression profile. In addition, the present gene-expression data set is further explored to obtain specific biological insights associated with the aggressive behaviour of this invasive type of breast cancer. This is of particular importance because no real progression has been made in treating this type of breast cancer, with the exception of a minor improvement due to the induction of taxanes. These novel insights might lead towards novel targets for treatment of breast tumours from patients with IBC.

## MATERIALS AND METHODS

### Patients and samples

Tumour samples were obtained from patients with breast adenocarcinoma treated in the Breast Clinic of the General Hospital Sint-Augustinus, Wilrijk, Belgium. Each patient gave written informed consent. This study was approved by the Local Institutional Review Board. All samples were stored in liquid nitrogen within 15 min after excision (median delay of 9 min). Breast tumour samples included 19 pretreatment samples of patients with IBC, diagnosed by strictly respecting the criteria of the American Joint Committee on Cancer (Singletary *et al*, 2002). The presence of tumour emboli was, as an isolated pathological finding, not sufficient for the diagnosis of IBC. Of the 40 non-IBC samples, 9 represented LABC (eight T3 and one T4), 14 samples represented T2 tumours and 17 represented T1 tumours. Clinicopathologic characteristics for the IBC and non-IBC are provided in Table 1.

### RNA extraction, microarray hybridisation and normalisation

RNA was extracted as described before using the RNeasy Mini Kit (Qiagen, Valencia, CA, USA). One T3 tumour and one T2 tumour was sampled twice to assess the reproducibility of the gene-expression profiling experiments. RNA from 61 samples was hybridised onto HGU 133 plus 2.0 Affymetrix chips in collaboration with the VIB Microarray Facility (O&N, UZ-Gasthuisberg, Leuven, Belgium). Four IBC samples have already been used in our previous genome-wide gene-expression analysis experiments (Van Laere *et al*, 2005). Perfect match (PM) fluorescence intensities were background-corrected, mismatch (MM)-adjusted, normalised and summarised to yield log2-transformed gene-expression data using the GCRMA algorithm. All normalisation procedures and subsequent data analyses have been performed using Bioconductor in R (www.bioconductor.org).

### Data analysis

To remove data noise and reduce data dimensionality, all genes with gene-expression data above log2 (100) in at least 25% of the arrays were filtered in resulting in a gene list of 18 182 informative genes. Global views of the variation in gene expression among the different breast cancer samples defined by the informative gene list were obtained using principal component analysis. Next, 250 informative genes having the greatest s.d. were selected to perform an unsupervised hierarchical complete linkage cluster analysis with the Euclidean distance as similarity metric.

The presence of the five different cell-of-origin breast cancer subtypes, originally described by Perou *et al* (2000) for non-IBC, was investigated in the present data set using a centroid-mediated classification algorithm. Detailed methodology is described in Van Laere *et al* (2006a). Briefly, the intrinsic gene list was mapped onto our informative gene list resulting in 347 informative and intrinsic genes. For each of the five cell-of-origin subtypes, we computed the typical expression profile for the 347 genes in the original Norway/Stanford data set (Sorlie *et al*, 2003), hereafter designated centroid. To classify our breast tumour samples, Pearson correlations were calculated between each sample in our series and each centroid based on the Norway/Stanford data set (Sorlie *et al*, 2003). A breast tumour sample was classified according to the highest correlation coefficient between its molecular profile and any of the calculated centroids. Unsupervised hierarchical complete linkage clustering and principal component analysis was applied to investigate the reliability of the subtype classification.

Our previously described IBC signature, consisting of 756 genes able to separate IBC from non-IBC in an unsupervised hierarchical clustering (Van Laere *et al*, 2005), was mapped onto the list of informative genes, identifying 739 common genes. Using these genes, principal component analysis and unsupervised hierarchical complete linkage clustering was performed to investigate common biological themes present in this data set. The global expression difference of the IBC signature between IBC and non-IBC was investigated using Goeman's global test (Goeman *et al*, 2004). To investigate if our IBC signature truly reflects the difference between IBC and non-IBC in a platform-independent manner, gene set enrichment analysis (GSEA) was performed (Subramanian *et al*, 2005). Statistical significance of the GSEA was assessed using 10 000 permutations.

Nuclear factor-*κ*B hyperactivation in IBC was investigated using 105 genes activated by NF-*κ*B, originally described by Loercher *et al* (2004) and used to demonstrate aberrant NF-*κ*B activation in squamous cell carcinoma. The same gene list was used by Chung *et al* (2006) to describe activation of NF-*κ*B in high-risk head and neck squamous cell carcinoma. The NF-*κ*B signature was mapped onto our informative gene list resulting in 93 common genes between the NF-*κ*B signature and our informative gene list. Nuclear factor-*κ*B activation in IBC was then investigated using unsupervised hierarchical complete linkage clustering, global testing and GSEA. Global testing and GSEA was additionally performed for gene lists representing several NF-*κ*B-related gene ontology (GO) identifiers. Statistical significance of the GSEA was assessed using 10 000 permutations.

Significance analysis of microarrays (SAM; Tusher *et al*, 2001) was performed to select genes with a differential gene-expression profile between IBC and non-IBC. Choosing a *δ*-value of 1.6, we selected 1794 genes with a false discovery rate (FDR) lower than 0.01. This gene list was intersected with a gene list containing all differentially expressed genes (FDR<0.05) from our previous data set, resulting in 115 common genes. Using the topGO algorithm (Alexa *et al*, 2006), these 115 common genes were analysed to identify which molecular functions are represented within this gene list. Significance for each individual GO-identifier was computed by applying Fisher's exact test on a weighted contingency table for the specified GO-identifier.

### Tissue microarray

Using the Beecher Instruments Tissue Arrayer (Beecher Instruments, Silver Springs, MD, USA) a tissue microarray (TMA) was constructed containing the same patient population as used for the genome-wide gene-expression profiling experiments. The TMA contained four core biopsies for every IBC and non-IBC patient. To minimise tissue loss during microtome sectioning and tissue transfer, the TMA was incubated for 30 min at 40°C. Five-micrometre slides were cut from the TMA and immunohistochemistry was performed for oestrogen receptor (ER), progesterone receptor (PR), Her2/neu oncogene (ErbB2), epidermal growth factor receptor (EGFR) and cytokeratin 5/6 (CK5/6). For ER, PR, ErbB2 and EGFR, the PharmDX system (Dako, Glostrub, Denmark) was used according to the manufacturer's instructions. For CK5/6 (clone D5-16 B4, Dako), TMA sections were rehydrated through sequential changes of alcohol and distilled water. Antigen retrieval was performed in Tris/EDTA (pH 9) during 30 min at 95°C. The antigen was incubated at room temperature at a concentration of 0.2 *μ*g ml^−1^. For ER and PR, a breast tumour was regarded positive when at least 10% of the tumour cell nuclei demonstrated protein expression. For ErbB2, scoring was performed according to the HercepTest scoring system. For EGFR and CK5/6, a tumour was regarded positive when respectively membranous and cytoplasmatic staining of tumour cells was observed.

### Statistical analysis

Statistical analysis other than microarray data analysis was performed using the SPSS 11.0. package (SPSS Inc., Chicago, IL, USA). Correlations between the clustering output and IHC data were calculated using a Pearson *χ*^2^ test or a Fisher's Exact test when appropriate. A *P*-value smaller than 0.05 was regarded as significant.

## RESULTS

### IBC and non-IBC show a distinct gene-expression profile

**Figure 1:**
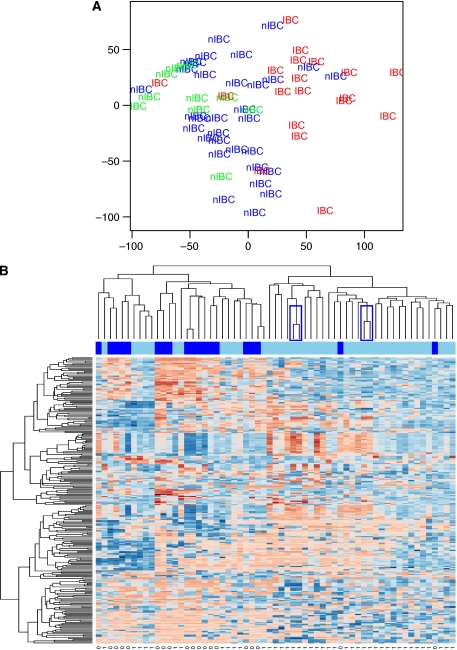
Principal component analysis using a list of 18.182 informative genes was performed to obtain global views of the variation in gene expression among the different breast cancer samples (**A**). Inflammatory breast cancer samples are colour-coded red, non-IBC T1 or T2 tumours are colour-coded blue and non-IBC T3 or T4 tumours are colour-coded green. The first principal component is represented by the *X*-axis, whereas the *Y*-axis represents the second principal component. Inflammatory breast cancer and non-IBC samples are separated along the first principal component. Unsupervised hierarchical complete linkage clustering was performed using 250 informative genes having the greatest s.d. (**B**). Gene-expression data for these 250 genes are represented in matrix format, with rows indicating genes and columns indicating samples. Overexpressed genes colour-coded red and underexpressed genes colour-coded blue. Colour saturation indicates the level of overexpression or repression. Tumour class is represented by a colour-coded bar underneath the dendrogram (IBC=dark blue; non-IBC=light blue). Replicate samples are indicated by blue rectangles. Two sample clusters have been identified, one cluster enriched in IBC specimens (17 out of 29) and other cluster enriched in non-IBC specimens (28 out of 30; Pearson *χ*^2^; *P*<0.0001).

Global views of the variation in gene expression among the different breast cancer samples defined by the informative gene list were obtained using principal component analysis. Inflammatory breast cancer (red) and non-IBC (green and blue) specimens are separated along the first principal component (*X*-axis; Figure 1A). The IBC specimens are scattered near the right end of the *X*-axis, whereas the non-IBC specimens scattered around the middle and near the left end of the *X*-axis. Strikingly, the global gene-expression pattern of IBC (red) specimens more closely resembled the global gene-expression pattern of T1 or T2 tumours (blue) instead of the global gene expression of LABC (T3 or T4 tumours, green).

Two hundred and fifty genes having the greatest s.d. were submitted to unsupervised hierarchical complete linkage clustering analysis. The clustering output is visualised in Figure 1B. Importantly, replicate RNA samples from the same breast tumours clustered on terminal branches, indicating the global reproducibility of our microarray experiment. For further statistical analysis, replicate samples are regarded as one sample. Two major sample clusters have been identified. A first sample cluster (left) is enriched in IBC specimens (17 out of 29), whereas a second sample cluster (right) was enriched in non-IBC specimens (28 out of 30; Pearson *χ*^2^, *P*<0.0001).

### Identification of the cell-of-origin subtypes in IBC and non-IBC

**Figure 2:**
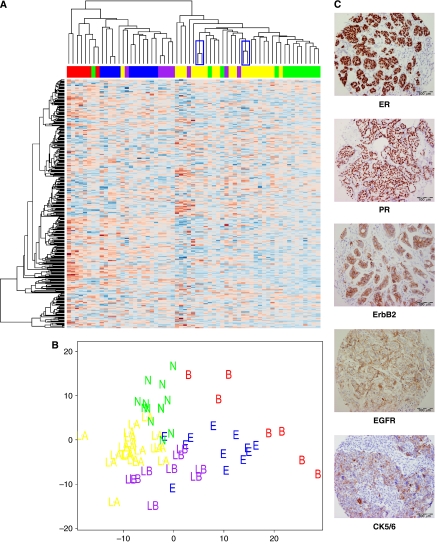
Unsupervised hierarchical complete linkage clustering was performed using 347 intrinsic genes (**A**). Gene-expression data for these 347 genes are represented in matrix format, with rows indicating genes and columns indicating samples. Overexpressed genes are colour-coded red and underexpressed genes are colour-coded blue. Colour saturation indicates the level of overexpression or repression. Branches of replicate samples are indicated using a blue rectangle. Cell-of-origin subtype classification for each breast tumour is indicated underneath the dendrogram. Cell-of-origin subtypes are colour-coded as follows: luminal A (yellow), luminal B (purple), normal-like (green), ErbB2-overexpressing (blue) and basal-like (red). Two sample clusters have been identified, one sample cluster containing most basal-like, ErbB2-overexpressing and luminal B samples (24 out of 27) and other sample cluster containing most luminal A and normal-like samples (30 out of 32; Pearson *χ*^2^; *P*<0.0001). Inflammatory breast cancer samples (16 out of 19) are contained within the first sample cluster and 30 out of 40 nIBC samples are contained within the second sample cluster (Pearson *χ*^2^; *P*<0.0001). Principal component analysis using the same gene set was performed (**B**) and similarly colour-coded. The distinct cell-of-origin subtype clusters are clearly visible, confirming our initial centroid-mediated classification. The ER+ cell-of-origin subtypes (luminal A, luminal B and normal-like) are separated from the ER− cell-of-origin subtypes (basal-like and ErbB2-overexpressing) along the *X*-axis (first principal component), implicating ER expression as the major discriminator within the intrinsic gene list. The classification was validated using IHC for ER, PR, ErbB2, EGFR and CK5/6 on a TMA containing core biopsies from the same patients as those used for the genome-wide gene-expression analysis. Microphotographs, visualising staining results are displayed in (**C**).

The presence of different breast cancer cell-of-origin subtypes has been repeatedly observed in IBC (Bertucci *et al*, 2005; Van Laere *et al*, 2006a). We have shown that IBC primarily segregates into the basal-like or ErbB2-overexpressing subgroups (Van Laere *et al*, 2006a). We have investigated the presence of the different cell-of-origin subtypes in the current data set using a centroid-mediated clustering algorithm. For further statistical analysis, replicate samples were regarded as one sample. Five out of seven (71%) basal-like, 8 out of 12 (67%) ErbB2-overexpressing, 1 out of 19 (5%) luminal A, 3 out of 8 (38%) luminal B and 2 out of 13 (15%) normal-like breast tumours were IBC samples. Altogether, 13 out of 19 IBC samples belonged to the combined basal-like and ErbB2-overexpressing subtype, whereas only 6 out of 40 non-IBC belonged to the combined basal-like and ErbB2-overexpressing cluster (Pearson *χ*^2^; *P*<0.0001). These classification results have been confirmed using unsupervised hierarchical complete linkage clustering analysis (Figure 2A) and principal component analysis (Figure 2B).

The classification of the breast tumour into the different cell-of-origin subtypes was validated using IHC for ER, PR, ErbB2, EGFR and CK5/6 using a TMA containing core biopsies of each breast tumour in our gene-expression data set. Microphotographs, demonstrating IHC staining results for each of the above-mentioned markers, are visualised in Figure 2C. Within the combined luminal A, luminal B and normal-like group, we identified 37 out of 40 breast tumour with ER protein expression, whereas in the remaining two subtypes, only 5 out of 19 breast tumours with ER expression were identified (Pearson *χ*^2^, *P*<0.0001). For PR, similar results were obtained. ErbB2 protein overexpression was found in 8 out of 12 breast tumours classified as belonging to the ErbB2-overexpressing subtype, whereas for the remaining subtypes only 4 out of 47 breast tumour displayed ErbB2 overexpression (Pearson *χ*^2^, *P*<0.0001). For the breast tumours classified as basal-like tumours, 5 out of 7 had no ER, PR or ErbB2 overexpression (‘triple negative’), whereas only 4 out of 52 non-basal-like breast tumours were triple negative (Pearson *χ*^2^, *P*<0.0001). In addition, 5 out of 7 and 4 out of 7 basal-like tumours showed respectively EGFR and CK5/6 overexpression compared to only 3 out of 52 and 5 out of 52 in the non-basal-like breast tumours (Pearson *χ*^2^, respectively *P*<0.0001 and 0.001).

### Platform-independent validation of our IBC signature

**Figure 3:**
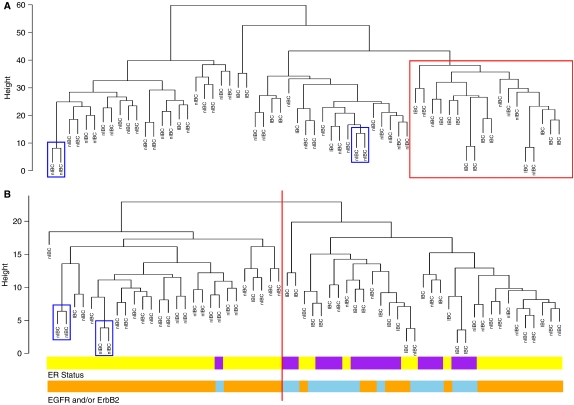
Unsupervised hierarchical complete linkage clustering was performed using a previously identified IBC signature. The resulting dendrogram is visualised (**A**). Tumour labels (IBC or nIBC) are printed immediately beneath each branch. The height of the branches, indicated in the *Y*-axis, is a measure for dissimilarity between clusters. Clusters generated at a higher level in the dendrogram are more dissimilar than clusters generated at a lower level in the dendrogram. Branches from replicate samples are indicated using a blue rectangle. One sample cluster, particularly enriched in IBC specimens (12 out of 18) is indicated using a red rectangle. The dendrogram resulting from unsupervised hierarchical complete linkage clustering, performed using an NF-*κ*B signature is visualised in (**B**). Again, tumour labels (IBC or nIBC) are printed immediately beneath each branch and replicate samples are indicated using a blue rectangle. One IBC-rich cluster (17 out of 33; right of the red line) and one IBC-poor (2 out of 26) cluster (left of the red line) was identified (Pearson *χ*^2^; *P*<0.0001). Oestrogen receptor status and combined EGFR and/or ErbB2 overexpression are indicated beneath the dendrogram using colour-coded bars (yellow: ER−; purple: ER+; orange: no EGFR or ErbB2 overexpression; light blue: EGFR and/or ErbB2 overexpression). The IBC-rich cluster contained 16 out of 33 ER− and 17 out of 33 EGFR and/or ErbB2+ breast tumour specimens compared to only 1 out of 26 ER− and 1 out of 26 EGFR and/or ErbB2+ breast tumour specimens in the IBC-poor cluster (Pearson *χ*^2^; respectively *P*<0.0001 and 0.0001).

Previously, we described an IBC signature, based on the expression of 756 genes able to separate IBC from non-IBC using a cluster analysis (Van Laere *et al*, 2005). To perform a platform-independent validation of our IBC signature, we submitted the present data set to unsupervised hierarchical complete linkage clustering using 739 genes in common between the IBC signature and the informative gene list. The resulting dendrogram is visualised in Figure 3A. We identified one sample cluster, mainly enriched in IBC specimens (12 out of 18). These results have been confirmed using principal component analysis (data not shown).

Next, we investigated if the IBC signature truly captures the difference between IBC and non-IBC. Therefore, we investigated if the genes, belonging to the IBC signature, were significantly overrepresented in the list of differentially expressed genes between IBC and non-IBC obtained using the present data set. Using the GSEA algorithm, we obtained a *P*-value of 0.048 for the IBC signature. Moreover, using the Goeman's global test, we demonstrated that the global expression pattern of the IBC signature is significantly related to the difference between IBC and non-IBC (*P*<0.0001).

### NF-*κ*B hyperactivation is a common feature in IBC

Previously, we have demonstrated that NF-*κ*B hyperactivation is a common feature of IBC (Van Laere *et al*, 2006b). To investigate NF-*κ*B hyperactivation in the current data set, we applied unsupervised hierarchical complete linkage clustering using an NF-*κ*B signature as input gene list (Loercher *et al*, 2004). The dendrogram is visualised in Figure 3B. Replicate samples were regarded as one sample. We identified two sample clusters, one sample cluster mainly enriched in non-IBC specimens (24 out of 26; blue), and one sample cluster containing nearly all IBC specimens (17 out of 33; grey; Pearson *χ*^2^, *P* 0.0002). The global expression pattern of the NF-*κ*B signature was linked to the difference between IBC and non-IBC (Goeman's global test, *P*=0.0003). However, genes belonging to the NF-*κ*B signature were not significantly overrepresented in the list of differentially expressed genes between IBC and non-IBC obtained using the current data set (GSEA, *P*=0.293). These results were confirmed using the same analyses for gene lists derived from NF-*κ*B-related GO identifiers (Table 2).

Interestingly, unsupervised hierarchical complete linkage clustering, using the NF-*κ*B signature as input gene list, performed better in separating ER+ from ER− breast tumour specimens. The IBC-rich sample cluster contains 16 out of 33 ER− breast tumour specimens, whereas the IBC-poor sample cluster contains only 1 out of 26 ER− breast tumour specimen (Pearson *χ*^2^, *P*<0.0001; Figure 3B). In addition, nearly all breast tumours having EGFR and/or ErbB2 overexpression were contained in the IBC-rich sample cluster: 17 out of 33 EGFR and/or ErbB2-positive samples in the IBC-rich cluster *versus* 25 out of 26 EGFR- and ErbB2-negative samples in the IBC-poor cluster (Pearson *χ*^2^, *P*<0.0001; Figure 3B).

### Gene ontology analysis of commonly overexpressed genes

**Figure 4:**
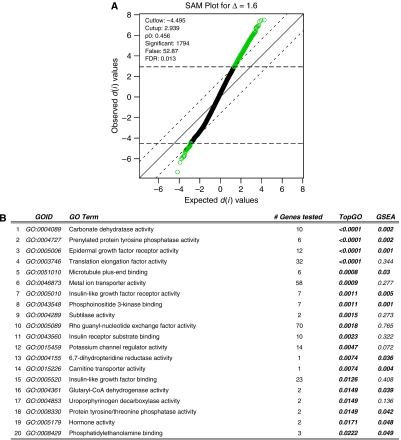
SAM plot generated using a *δ*-value of 1.6 to control the FDR at a level of 0.01 (**A**). Totally, 1794 genes are significantly different in expression between IBC and non-IBC, from which approximately 53 genes are expected to be false positives. This list of 1794 was intersected with a list of differentially expressed genes from an earlier study, resulting in 115 commonly overexpressed genes. This gene list was then analysed using the topGO algorithm to identify molecular functions overrepresented within this gene list. The 20 top-ranked molecular functions are represented in (**B**). For each GO identifier, we reported the number of genes tested, as well as the *P*-value for both topGO analysis and GSEA.

To gain deeper insights into the biology of IBC, we selected 1794 differentially expressed genes using SAM (Figure 4A) and intersected this gene list with differentially expressed genes obtained from the former data set. We identified 115 commonly overexpressed genes in either IBC or non-IBC. The 20 top-ranked molecular functions, significantly overrepresented in the gene list of commonly overexpressed genes, are listed in Figure 4B. Among the 20-top ranked GO identifiers related to molecular functions, 3 GO identifiers were related to insulin-like growth factor signalling (GO:0005010, GO:0005520, GO:0043560). Gene set enrichment analysis showed that genes belonging to GO:0005010 are significantly overrepresented among the differentially expressed genes (Figure 4B). In addition, ‘phosphoinositide 3-kinase binding’ (GO:0043548), a molecular function implicated in PI3K signalling, was identified.

## DISCUSSION

Inflammatory breast cancer is a specific and aggressive form of LABC, among others characterised by an elevated metastatic and angiogenic potential (Van der Auwera *et al*, 2004). A better understanding of the specific biology of the breast tumours associated with IBC might lead to improved therapeutic modalities as well as to new and sensitive diagnostic tests. In addition, unravelling the biological processes involved in the efficient metastatic spread, characteristic of IBC tumour cells, might lead to a better understanding of the mechanisms behind breast cancer metastasis in general. Therefore, efforts were undertaken to gain deeper insights at molecular level into the biology of IBC. Hence, it was demonstrated that IBC shows a distinct gene-expression profile compared to non-IBC (Bertucci *et al*, 2004; Van Laere *et al*, 2005). In the present study, this observation was confirmed in a platform-independent manner, using both principal component analysis and unsupervised hierarchical clustering. The greatest variation in gene expression in the present data set is attributable to the distinction between IBC and non-IBC. Strikingly, the gene-expression profile of IBC tumours more closely resembles the gene-expression profile of T1 or T2 tumours instead of T3 or T4 tumours, which might be explained by the fact that IBC tumours are quickly arising tumours, instead of longstanding tumorigenic processes. In fact, this observation argues in favour of a non-stage-matched approach when comparing IBC to non-IBC. The identification of an IBC-specific gene-expression profile suggests that some biological processes might be associated with the clinical presentation discriminating IBC from non-IBC. Since it has been proven that the tumour stroma-associated gene-expression profile significantly contributes to the overall gene-expression profile of breast tumours (Yang *et al*, 2006), we cannot exclude that tumour/host interactions play an important role in the clinical presentation of IBC. Hence, it is, at this point, impossible to say whether the different gene-expression profile of IBC reflects a different gene-expression profile of IBC tumour cells, a different constitution of the tumour-associated stroma or both.

As shown previously, the differences in gene expression, observed between IBC and non-IBC, are, in part, attributable to the differential presence of the different cell-of-origin breast cancer subtypes. Referring to our initial data set, breast tumours from patients with IBC showed a higher propensity of belonging to the basal-like or ErbB2-overexpressing cell-of-origin subtype (Van Laere *et al*, 2006a). Given the frequent ErbB2 and/or EGFR overexpression and the frequent loss of ER protein expression in IBC, as well as the reduced overall survival for patients with IBC compared to patients with non-IBC, this observation could not be regarded as a coincidence. In the same study, however, we also demonstrated that the specific gene-expression profile of breast tumours from patients with IBC is not only attributable to the preferential segregation of these breast tumours in the basal-like or ErbB2-overexpressing breast cancer subtypes. In fact, only a small percentage of the specific gene-expression profile of breast tumours from patients with IBC is cell-of-origin subtype-specific (Van Laere *et al*, 2006a). Using a centroid-mediated classification algorithm to analyse the presence of the different cell-of-origin breast cancer subtypes in the present data set, we again showed that breast tumours from patients with IBC significantly more often belong to the basal-like or ErbB2-overexpressing cell-of-origin subtypes. The classification of the breast tumour samples into the different cell-of-origin subtypes was in close agreement with IHC staining results obtained for ER, PR, ErbB2, EGFR and CK5/6, thereby validating our classification. As mentioned previously, the significant overrepresentation of the basal-like or the ErbB2-overexpressing cell-of-origin subtype in the group of breast tumours from patients with IBC cannot fully explain the characteristics associated with IBC, since several IBC specimens clearly show a luminal A, luminal B or normal-like phenotype. In addition, most basal-like or ErbB2-overexpressing breast tumours do not present the same clinical characteristics associated with IBC.

The distinct gene-expression profile of breast tumours from patients with IBC was translated into an IBC signature, based on the expression of 756 genes, able to separate IBC from non-IBC in an unsupervised hierarchical clustering analysis (Van Laere *et al*, 2005). In the present study, we demonstrate that the IBC signature truly captures the difference between IBC and non-IBC, since genes belonging to the IBC signature are significantly overrepresented in a list of differentially expressed genes generated in a platform-independent manner, as shown by GSEA. One of the hallmarks of our IBC signature was the presence of numerous NF-*κ*B target genes, suggesting NF-*κ*B hyperactivation in IBC (Van Laere *et al*, 2005, 2006b). In the present data set, the hyperactivation of NF-*κ*B in breast tumours from patients with IBC was investigated using an NF-*κ*B signature, described by Loercher *et al* (2004). Cluster analysis and global testing demonstrated that NF-*κ*B hyperactivation was indeed associated with IBC, but, despite this association, NF-*κ*B hyperactivation is not the main causative molecular alteration in IBC, as shown by GSEA. Hence, NF-*κ*B hyperactivation is not specific for IBC, which agrees with previous findings, that NF-*κ*B hyperactivation is implicated in the generation ER-negative breast cancer in general (Biswas *et al*, 2001; Zhou *et al*, 2005; Van Laere *et al*, 2007). One possible explanation for the involvement of NF-*κ*B in ER-negative breast cancer is the hyperactivation of MAP kinases secondary to EGFR and/or ErbB2 overexpression, leading to an NF-*κ*B-dependent downregulation of ER expression (Van Laere *et al*, 2007). In this context, we have shown that the NF-*κ*B signature performed better in separating ER-negative breast tumours from ER-positive breast tumours as well as EGFR- and/or ErbB2-overexpressing breast tumours from their EGFR-negative and ErbB2-negative counterparts. Hence, with respect to NF-*κ*B activation, IBC is not a distinct entity but merely constitutes a minor part of the spectrum of ER-negative breast tumours.

To gain deeper insight into the mechanisms active in breast tumours from patients with IBC, we intersected the list of differentially expressed genes from the former and the present study. Hence, we identified 115 commonly overexpressed genes that were analysed to investigate which molecular functions are represented within this gene list. Different GO-identifiers, linked to insulin-like growth factor (IGF) signalling were identified. The IGF pathway has been implicated in cell motility and breast cancer metastasis (Zhang *et al*, 2005), both major hallmarks of IBC. Moreover, loss of IGF-binding protein-related protein (IGFBP-rP9) is observed in 90% of the IBC specimens (Van Golen *et al*, 1999), which leads to increased IGF signalling and activation of RhoC (Kleer *et al*, 2004). In cell line experiments, insulin-like growth factor 1 (IGF1) activates Rho GTPases and IGF1-stimulated cell motility requires activation of PI3K (Zhang *et al*, 2005). Interestingly, genes belonging to ‘phosphoinositide 3-kinase binding’ and ‘Rho guanyl-nucleotide exchange factor activity’, molecular functions implicated in respectively PI3K signalling and Rho GTPase activation, are overrepresented in the list of commonly overexpressed genes. These data are in agreement with previously published data on IBC. Altogether, our gene-expression data provide the fingerprints of a pro-metastatic-signalling pathway, potentially explaining the highly invasive and metastatic phenotype of IBC.

## Figures and Tables

**Table 1:** Clinicopathological characteristics for IBC and non-IBC patients

	**Data set (*n*=59)**		
	**Non-IBC (*n*=40)**	**IBC (*n*=19)**	***P***
*Age (years)*			
Median (range)	60 (31–89)	63 (45–78)	0.319
			
*Histological type*			
Ductal	34	17	0.639
Lobular	6	2	
			
*Tumour emboli in dermal lymph vessels*			
Present	9	15	<0.0001
Absent	31	4	
			
*Grade* a			
1	16	0	0.003
2	15	9	
3	9	10	
			
*T-stadium*			
1	17	0	<0.0001
2	14	0	
3	8	0	
4	1	19	
			
*N-stadium* b			
0	18	1	0.002
1	12	5	
2	10	13	
			

IBC=inflammatory breast cancer.

a According to the Elston–Ellis modification of the SBR grading system.

b The N-stadium for patients with IBC was determined clinically.

**Table 2:** Global test and GSEA on gene lists of NF-*κ*B related GO-identifiers

	**GO ID**	**GO term**	**No. of genes tested**	**Global test**	**GSEA**
1	GO:0004704	NF-*κ*B-inducing kinase activity	2	0.1212	0.662
2	GO:0007249	I-*κ*B kinase/NF-*κ*B cascade	169	**<0.0001**	0.624
3	GO:0007250	Activation of NF-*κ*B-inducing kinase	13	**0.0001**	0.177
4	GO:0007253	Cytoplasmic sequestering of NF-*κ*B	6	0.4838	0.815
5	GO:0008588	Release of cytoplasmic sequestered NF-*κ*B	2	**0.0175**	0.737
6	GO:0042345	Regulation of NF-*κ*B import into nucleus	9	**0.0207**	0.847
7	GO:0042346	Positive regulation of NF-*κ*B import into nucleus	3	**0.0182**	0.92
8	GO:0042347	Negative regulation of NF-*κ*B import into nucleus	6	0.4838	0.822
9	GO:0042348	NF-*κ*B import into nucleus	9	**0.0207**	0.837
10	GO:0043122	Regulation of I-*κ*B kinase/NF-*κ*B cascade	131	**<0.0001**	0.448
11	GO:0043123	Positive regulation of I-*κ*B kinase/NF-*κ*B cascade	121	**0.0001**	0.693
12	GO:0043124	Negative regulation of I-*κ*B kinase/NF-*κ*B cascade	8	**<0.0001**	**0.009**
13	GO:0051059	NF-*κ*B binding	9	0.3102	0.712
14	GO:0051092	Activation of NF-*κ*B transcription factor	13	**0.0001**	0.509

GSEA=gene set enrichment analysis; GO=gene ontology; NF-*κ*B=nuclear factor-kappaB.
